# Identification of Neuropeptide F (NPF) Signaling and Associated Regulation of Food Intake in the Dark Black Chafer Beetle *Holotrichia parallela*

**DOI:** 10.3390/biology15120903

**Published:** 2026-06-09

**Authors:** Yang Chen, Huihui Hu, Wenjie Li, Xuanling Wei, Long Du, Dongdong Tian, Mingjing Qu, Zhongjun Gong, Xiao Li, Yongsheng Yao

**Affiliations:** 1 College of Agriculture, Tarim University, Aral 843300, China; ydz2111@163.com (Y.C.); zbhh016@163.com (H.H.); liwenjie010719@163.com (W.L.); m15599940597@163.com (X.W.); 2 Shandong Peanut Research Institute, Qingdao 266100, China; dulongzxcv@163.com (L.D.); tiandd0101@163.com (D.T.); mjqu2013@163.com (M.Q.); 3 College of Plant Protection, Henan Agricultural University, Zhengzhou 450002, China

**Keywords:** *Holotrichia parallela*, neuropeptide F, neuropeptide F receptor, RNA interference

## Abstract

*Holotrichia parallela* is a globally distributed soil-dwelling pest that poses a major threat to peanut cultivation in China. Neuropeptides, as critical signaling molecules, regulate multiple physiological and behavioral processes in insects and represent highly promising targets for pest management. To date, the functional characteristics of neuropeptides in *H. parallela* remain unreported. In this study, we isolated and cloned one *NPF* and one *NPFR* gene, respectively. Bioinformatics analysis revealed that alternative splicing of the *NPF* gene produces two transcript variants, NPFa (255 bp) and NPFb (369 bp). The *NPFR* gene spans a length of 1188 bp, encoding 395 amino acids that contain seven α-helical transmembrane domains, indicating that it belongs to the family A G protein-coupled receptor (GPCR) family. Spatiotemporal expression profiles demonstrated that *NPF* was most abundant in the adult brain, whereas *NPFR* was highly enriched in the brain and antennae. *NPF* expression peaked in second-to-third-instar larvae, while *NPFR* was highly expressed in eggs. Starvation stress significantly upregulated the expression of both genes. RNA interference (RNAi)-mediated silencing of *NPF* and *NPFR* significantly reduced food intake, female fecundity, and glycogen content in adults. These findings enhance our understanding of insect neuropeptides signaling networks and support the development of behavior-based pest control strategies.

## Simple Summary

*Holotrichia parallela* Motschulsky is a major worldwide subterranean pest, causing severe damage to multiple crops, especially peanuts in China. For decades, chemical insecticides have served as the primary method for controlling this pest, leading to significant challenges including insecticide resistance and food safety risks associated with pesticide residues. Consequently, there is an urgent need to identify safe and effective alternatives for pest management. Neuropeptides are critical regulators of various physiological processes in insects and their receptors represent potential targets for pest control. However, the functional roles of a specific neuropeptide F (*NPF*) and neuropeptide F receptor (*NPFR*) in *H. parallela* remain poorly understood. In this study, we investigated the functions of *NPF* and *NPFR* and their responses to different environmental stressors. Our findings demonstrate that *NPF* and *NPFR* play a crucial role in regulating feeding behavior and energy metabolism.

## 1. Introduction

Insect neuropeptides are trace peptide compounds secreted by neurosecretory glands or neurosecretory cells, representing the most diverse signaling molecules in insects. They are released and function as hormones or neurotransmitters [1,2]. These neuropeptides are evolutionarily conserved and participate in a wide range of physiological processes [3]. Neuropeptides exert their physiological functions by binding to their respective receptors, thereby activating downstream signaling pathways. With few exceptions, most insect neuropeptide receptors belong to the G protein-coupled receptor (GPCR) family [4]. The insect neuropeptide signaling system is considered an ideal target for developing environmentally friendly pest control agents, offering novel approaches for sustainable insect management [5]. Among the diverse insect neuropeptides, NPF is one of the most extensively studied members, exhibiting versatile physiological roles.

NPF, a member of the insect neuropeptide family, is named for the C-terminal phenylalanine (F) of its mature peptide and is the homolog of vertebrate neuropeptide Y(NPY) [6]. Mature NPF typically consists of 28–45 amino acids with a conserved C-terminal RxRFamide motif and is primarily secreted by cells in the central and peripheral nervous systems [7]. The first authentic insect NPF was identified in *Drosophila melanogaste* [8]. Subsequently, NPF has been identified in dozens of insect species, including the dipterans *Aedes aegypti* [9] and *Anopheles gambiae* [10]; the hymenopteran *Apis mellifera* [11]; the lepidopterans *Bombyx mori* [12] and *Helicoverpa zea* [13]; the orthopteran *Locusta migratoria* [14]; the isopteran *Reticulitermes flavipes* [15]; and the coleopterans *Rhynchophorus ferrugineus* [16] and *Dendroctonus armandi* [17]. NPF modulates diverse physiological and behavioral processes by binding to its receptor (NPFR) and activating downstream effectors. The most extensively studied function of NPF is feeding regulation, including food intake and preference [18,19,20,21,22,23,24], though its roles vary across insect species. The underlying mechanisms may involve interactions with other neurohormones or signaling pathways [25]. NPF has also been reported to regulate molting and metamorphosis [26], as well as multiple physiological and behavioral processes such as olfaction [27], learning and memory [28], stress responses and locomotion [29]. Furthermore, the NPF signaling system is involved in regulating alcohol sensitivity [18], circadian rhythms [29,30,31], aggressive behavior [32] and responses to high-temperature stress [33]. From a potential application perspective, the NPF/NPFR signaling pathway could be exploited to regulate insect feeding, metabolism, reproduction, or behavior through methods such as peptide mimetics, receptor agonists/antagonists, or RNAi-mediated interference with ligand or receptor expression. Therefore, functional characterization of the NPF/NPFR signaling system in *H. parallela* serves as a functional proof-of-concept for regulatory targets.

The dark black chafer *H. parallela* is a destructive subterranean pest. Its larvae (commonly referred to as grubs) inhabit the soil and feed on the roots and fruits of various crops such as peanuts, corn, and sweet potatoes. The adults display an unusual 48 h periodicity (circabidian rhythm) in certain physiological processes and behaviors [34]. Specifically, they remain in the soil during daylight hours and emerge aboveground for mating and feeding shortly after sunset every other night. Currently, the application of chemical insecticides remains the primary approach for controlling grubs. However, the overuse of chemical insecticides has led to numerous problems, including pesticide residues, biodiversity loss, and insecticide resistance [35,36,37].

Feeding motivation, energy reserves, and reproductive investment in insects are generally regulated by the neuroendocrine system [1]. Although the NPF signaling pathway has been studied in a growing number of insect species, the molecular identity and physiological functions of NPF and its receptor NPFR in *H. parallela* remain unclear. Therefore, the scientific question addressed in this study is not merely the lack of genetic annotation of NPF/NPFR in this species but rather whether a conserved neuropeptide pathway involved in feeding regulation functions in a beetle that exhibits a bi-daily pattern of feeding and mating. Based on the conserved role of NPF in regulating insect feeding, we hypothesize that the NPF/NPFR signaling system in *H. parallela* is involved in the regulation of feeding and energy metabolism. Given the bi-daily rhythm of adults, we further speculate that NPF signaling may also be associated with species-specific physiological adaptations related to periodic feeding, energy allocation, and reproduction. *H. parallela* displays a distinct bi-daily behavioral rhythm, unlike the typical daily circadian rhythm of most insects. Deciphering the NPF regulatory pattern in *H. parallela* and performing a comparative analysis of its similarities and differences with the canonical pathways in diurnal insects will bridge a significant knowledge gap in the regulatory mechanisms of insects with bimodal rhythms, thereby holding considerable scientific value.

In this study, we identified and characterized *NPF* and its candidate receptor NPFR in *H. parallela*. The innovative aspects of this research include the first molecular identification of the NPF/NPFR signaling system in this species, as well as the discovery of two NPF transcript variants, *NPFa* and *NPFb*. We further analyzed their sequence characteristics, phylogenetic relationships, developmental stage- and tissue-specific expression patterns, and their transcriptional responses to starvation stress. Finally, we employed RNA interference to examine whether NPF/NPFR signaling is involved in regulating feeding, fecundity, and glycogen metabolism. By integrating descriptive molecular identification with functional RNAi experiments, this study aims to determine whether the NPF/NPFR signaling pathway plays a conserved feeding-related role in *H. parallela* and to provide a foundation for future investigations into its potential species-specific functions in bi-daily rhythmic physiology.

## 2. Materials and Methods

### 2.1. Insect and Sample Collection

Adult *H*. *parallela* specimens were collected from peanut fields in Qingdao, China (36°48′46″ N, 120°30′17″ E) during their emergence from the soil throughout the flight seasons (June–August 2023–2024). In the laboratory, the insects were maintained in plastic containers (48 × 35 × 30 cm) containing an approximately 20 cm layer of a 12:1 sieved loam-sand mixture (30-mesh sieve) at 18–20% humidity. Adults were provided with fresh elm (*Ulmus pumila*) leaves daily, while wheat (*Triticum aestivum* L.) seedlings were cultivated in larval rearing containers to facilitate root feeding by larvae. Rearing conditions were maintained at 25 ± 1 °C, 70% relative humidity, and a 14L:10D photoperiod.

### 2.2. Full-Length Cloning

Using amino acid sequences of NPF and NPFR from *Tribolium castaneum* [38], *R. ferrugineus* [16] and *Dendroctonus ponderosae* [17] as query sequences, a homologous search was performed to identify NPF precursor and its receptor from a head transcriptome dataset of *H. parallela* (unpublished) via local BlastP alignment with an E-value threshold set at ≤10^−5^. This analysis identified a unigene containing the complete open reading frame (ORF) of NPF and another unigene encoding a partial sequence of NPFR, designated as *H. parallela NPF* and *NPFR*, respectively.

The sequences were then verified through RT-PCR and Sanger sequencing. Total RNA was isolated from the head samples of *H. parallela* adults using RNAiso Plus (TaKaRa Biotech, Dalian, China) and synthesized into single-strand cDNA as a PCR template with the RevertAid First Strand cDNA Synthesis Kit (Thermo Scientific, Waltham, MA, USA). Gene-specific primers for RT-PCR were designed using Primer-BLAST (2.17.0, https://blast.ncbi.nlm.nih.gov/Blast.cgi, accessed on 9 February 2026). to amplify the full-length ORF of NPF and the partial sequence of NPFR (Table S1). The PCR products were separated by 1.0% agarose gel electrophoresis, and target bands were excised and purified using a gel extraction kit. The purified amplicons were subcloned into the pMD^TM^19-T Vector Cloning Kit (TaKaRa Biotech, Dalian, China) and sequenced by Sangon Biotech Co., Ltd. (Shanghai, China).

To obtain the full-length cDNA sequence of *NPFR*, 5′ and 3′ rapid amplification of cDNA ends (RACE) were performed using the RACE kit from (Sangon Biotech, Shanghai, China). Specific primers for 5′ and 3′ RACE were designed based on the obtained NPFR sequence (Table S1). To enhance amplification specificity, touchdown PCR was employed for the 5′- and 3′-untranslated regions (UTRs) with an annealing temperature gradient of 65–55 °C. The amplified products were subsequently cloned and sequenced by the Sanger method as described above.

### 2.3. Bioinformatic Analysis of NPF

The ORFs of *NPF* and *NPFR* genes were predicted online using ORF Finder (https://www.ncbi.nlm.nih.gov/orffinder/, accessed on 9 February 2026). Physicochemical properties, including molecular weight (MW) and theoretical isoelectric point (pI), were analyzed using ProtParam (https://web.expasy.org/protparam/, accessed on 9 February 2026). Homology searches were performed via BLASTP on NCBI (https://blast.ncbi.nlm.nih.gov/Blast.cgi, accessed on 9 February 2026). Signal peptides in the NPF precursor were predicted using SignalP 6.0 Server (https://services.healthtech.dtu.dk/services/SignalP-6.0/, accessed on 9 February 2026), and mature peptides were predicted with NeuroPred (http://stagbeetle.animal.uiuc.edu/cgi-bin/neuropred.py, accessed on 9 February 2026) [39]. Transmembrane domains of the NPFR protein were analyzed using TMHMM 2.0 Server (http://www.cbs.dtu.dk/services/TMHMM-2.0/, accessed on 9 February 2026). The amino acid sequences used for alignment and phylogenetic analysis were retrieved from NCBI GenBank (https://www.ncbi.nlm.nih.gov/genbank/, accessed on 9 February 2026) or obtained from the previously published literature for entries without accession numbers (detailed information is provided in Supplementary Table S1) [17,40]. Multiple sequence alignments of amino acids were conducted using Clustal W (https://www.genome.jp/tools-bin/clustalw, accessed on 9 February 2026) [41], and edited with Jalview, and the sequence conservation was graphically represented by Weblogo (https://weblogo.berkeley.edu/, accessed on 9 February 2026) [42]. Sites of N-glycosylation and phosphorylation were predicted using NetNGlyc 1.0 (https://services.healthtech.dtu.dk/services/NetNGlyc-1.0/, accessed on 9 February 2026) and NetPhos 3.1 (https://services.healthtech.dtu.dk/services/NetPhos-3.1/, accessed on 9 February 2026), respectively. Phylogenetic trees were constructed using the neighbor-joining method in MEGA 11 (https://www.megasoftware.net/, accessed on 9 February 2026) with 1000 bootstrap replicates [43].

### 2.4. RNA Extraction and Quantitative RT-qPCR Analysis

Total RNA was extracted from the head samples of adult *H. parallela* using TransZol Up Plus RNA reagent (TransGen Biotech, Beijing, China). Genomic DNA was removed from total RNA using HiScript^®^ III RT SuperMix for qPCR (+gDNA wiper) (Vazyme Biotech, Nanjing, China). RNA concentration and purity were measured using a NanoDrop 2000 spectrophotometer (Thermo Fisher Scientific, Waltham, MA, USA), with the optical density (OD) A260/A280 ratio maintained between 1.8 and 2.0.

Reverse transcription-quantitative PCR (RT-qPCR) was performed on an ABI Prism 7500 Fast Detection System (Applied Biosystems, Foster City, CA, USA) following the manufacturer’s instructions for the SYBR Green Premix Pro Taq HS qPCR Kit (Accurate Biotechnology, Changsha, China). The reaction mixture (20 μL) consisted of 5 μL 10-fold diluted cDNA template, 0.4 μL of each primer (10 μM), 0.4 μL Rox, 10 μL SYBR Green Pro Taq HS and 3.8 μL nuclease-free water. PCR conditions were as follows: 95 °C for 30 s, followed by 40 cycles of 95 °C for 5 s and 60 °C for 30 s. Melting curve analysis was conducted from 65 °C to 95 °C. Three biological replicates and three technical replicates were performed for each condition. Relative expression levels were calculated using the 2^−ΔΔCt^ method [44].

### 2.5. Stage- and Tissue-Specific Analysis of NPF and NPFR

RT-qPCR was used to quantify the expression levels of target genes across different developmental stages and adult tissues of *H. parallela*. Female and male 45-day-old adults were dissected under a stereomicroscope in pre-chilled 0.1 M PBS buffer. For each biological replicate, brains, foreguts, midguts, hindguts, Malpighian tubules, ovaries, and testes were dissected from 10 individuals (pooled by sex). For antennae, 40 antennae (from 20 individuals of each sex) were collected per replicate. Three or four biological replicates were prepared for each tissue type.

For stage-specific analysis, adult insects emerging from the same field plot on the same day were collected and maintained in rearing boxes at a 2:1 female-to-male ratio for at least two weeks. Subsequently, females were transferred to a separate rearing box for oviposition and were removed after 24 h to synchronize the developmental stages of the offspring. Samples representing different developmental stages included 3-day-old eggs, 10-day-old first-instar larvae (Larvae I), early (Larvae IIa) and late (Larvae IIb) second-instar larvae, 30-day-old third-instar larvae, 7-day-old early pupae and 12-day-old late pupae (designated as Pupa a and Pupa b groups), as well as 45-day-old adults. For each developmental stage, biological replicates were prepared as follows: 10 eggs, 5–7 larvae, 3–4 pupae, or 3–4 adults per replicate. Three replicates were prepared for each group, except the late pupal stage (*n* = 2).

Two housekeeping genes, *GAPDH* (GenBank Accession No. MW661066.1) and Actin (GenBank Accession No. MT991084), were used as internal reference genes [45,46]. RT-qPCR primers (Table S1) were designed using Primer-BLAST. The specificity of each primer pair was validated by melting curve analysis and 1% agarose gel electrophoresis, and amplification efficiencies were evaluated using a standard curve generated from a five-point 1:5 dilution series of cDNA.

### 2.6. Effects of Stress Conditions on NPF and NPFR Expression

To evaluate the effects of heat stress, adult *H. parallela* reared at 25 °C were transferred to 36 °C or 39 °C for 3 h, with control groups maintained at 25 °C. For each treatment and control group, 20 female and 20 male surviving insects were collected separately. For females, three biological replicates were prepared, each pooling 6–7 heads. For males, four biological replicates were prepared, each pooling 5 heads. To assess the impact of starvation, adult *H. parallela* were separated by sex, then divided into two groups: one group was starved for 96 h, while the control group was provided with fresh elm leaves ad libitum. Twenty insects were used per sex per treatment, with five individuals pooled to form one biological replicate (four replicates in total). Total RNA was also extracted from dissected adult heads. RNA extraction, cDNA synthesis, and RT-qPCR were performed as described above.

### 2.7. dsRNA-Mediated Knockdown of NPF and NPFR

Double-stranded RNA (dsRNA) targeting *NPF* (dsNPF, 215 bp), *NPFR* (dsNPFR, 457 bp), and *GFP* (dsGFP, 472 bp; as a control) was synthesized in vitro using the T7 RiboMAX™ Express RNAi System (Promega, Madison, WI, USA), following the manufacturer’s instructions. The primers (Table S1) were designed by the online program SnapDragon-dsRNA Design (https://www.flyrnai.org/cgi-bin/RNAi_find_primers.pl, accessed on 9 February 2026). The quality of the purified dsRNA was assessed by the NanoPhotometer P-360 (Implen GmbH, Munich, Germany) and 1.0% agarose gel electrophoresis. The dsRNA products were diluted to 2 μg/μL with nuclease-free water and stored at −80 °C until use.

Prior to injection, 20- to 40-day-old adult beetles were anesthetized on ice for 5 min, followed by intrahemocoelic injection of 1 μL dsRNA (2 μg) through the intersegmental membrane between abdominal segments IV and V using a Hamilton 701 10 μL microsyringe (Hamilton, Reno, NV, USA). Beetles injected with equivalent volume of ds GFP served as controls. All injections were performed during the active phase of the beetles at scotophase (3:00 p.m.). After injection, beetles were maintained under standard rearing conditions as described above.

To assess the silencing efficiency, total RNA was extracted separately from the head and midgut tissues of adult *H. parallela* at 48, 96, 144, and 192 h post-injection. Each biological replicate consisted of pooled tissues from 10 individuals, with three independent replicates performed per treatment. The relative transcript levels of *NPF* and *NPFR* were quantified using RT-qPCR, as previously described.

### 2.8. Feeding Consumption Assay

Following dsRNA injection, food consumption assays were conducted. Each treatment group contained no fewer than 30 insects, reared individually. Both the ds*GFP* control group and the ds*NPF*/ds*NPFR* treatment groups were provided with an excess of fresh elm leaves, which were replaced daily. The body weight of each beetle and the remaining leaf quantity were measured daily at the time of injection using an electronic balance (precision = 0.0001 g, BSA124-S; Sartorius Ltd., Beijing, China). A blank control group without beetles was established to account for changes in leaf weight due to water content variation. Food consumption was calculated using the following formula [23,45]:

$$I\;=\;W-(L\;+\;\frac{\mathrm{aW}\;+\;\mathrm{bL}}{2})$$

$$a=\frac{M-N}{M}$$

$$b=\frac{M-N}{N}$$

*I* is the food consumption;

*W* is the initial weight of leaves in the experimental group;

*L* is the final weight of leaves in the experimental group;

*M* is the initial weight of leaves in the blank control group;

*N* is the final weight of leaves in the blank control group.

### 2.9. Survival and Reproduction Assays

Following dsRNA injection, survival and reproduction assays were conducted simultaneously using 20 pairs of adult beetles per treatment group. Each pair was reared in a small plastic box (20 cm× 20 cm× 25 cm) filled with an approximately 20 cm layer of loam-sand mixture under the conditions described above. The numbers of deaths and newly laid eggs in each box were recorded daily for 10 consecutive days after injection, with dead individuals and eggs removed promptly after each recording.

### 2.10. Determination of Glycogen, Free Fatty Acid, and Trehalose

To investigate the role of *NPF* and its receptor gene in energy metabolism of *H. parallela* adults, we first measured the levels of three energy metabolism indicators (glycogen, trehalose, and free fatty acid, FFA) in male and female adults under starvation conditions. Subsequently, these metabolic parameters were assessed at 96 h post-dsRNA injection. Whole-body homogenates from each group were utilized for the measurements. Glycogen and trehalose levels were quantified using the anthrone colorimetric method with commercial assay kits (Nanjing JC Dtech Biotechnology Co., Ltd., Nanjing, Jiangsu, China). Free fatty acids were measured using the FFAD-1-W kit (Suzhou Comin Biotechnology Co., Ltd., Suzhou, Jiangsu, China). Each measurement was performed in four biological replicates (five adults per replicate) using an Infinite 200 Pro microplate reader (TECAN, Männedorf, Switzerland).

### 2.11. Data Analysis

All statistical analyses were performed using GraphPad Prism 10.1.2 (GraphPad Software, San Diego, CA, USA). Data are presented as mean ± SE. Normality and homogeneity of variances were assessed using Shapiro–Wilk and Brown–Forsythe tests, respectively; both assumptions were met for all datasets (*p* > 0.05). For two-group comparisons, Student’s *t*-test was used. To correct for multiple comparisons across multiple *t*-tests, the Holm–Šídák (Holm) method was applied and an adjusted *p* value (*P*_adj_) < 0.05 was considered significant. For multi-group comparisons, one-way ANOVA was used, followed by Tukey’s test (spatiotemporal expression profile) or Dunnett’s test (RNAi-related assays); adjusted *p* values are reported (α = 0.05). Survival data were analyzed by Kaplan–Meier with the log-rank test. Regarding small sample sizes: in the stage-specific expression analysis, the late pupal group had only two biological replicates (*n* = 2); this group is reported descriptively without inferential statistics. Graphs were generated using GraphPad Prism 10.1.2.

## 3. Results

### 3.1. Molecular Characterization and Bioinformatic Analysis of NPF and NPFR Genes

**Figure 1:**
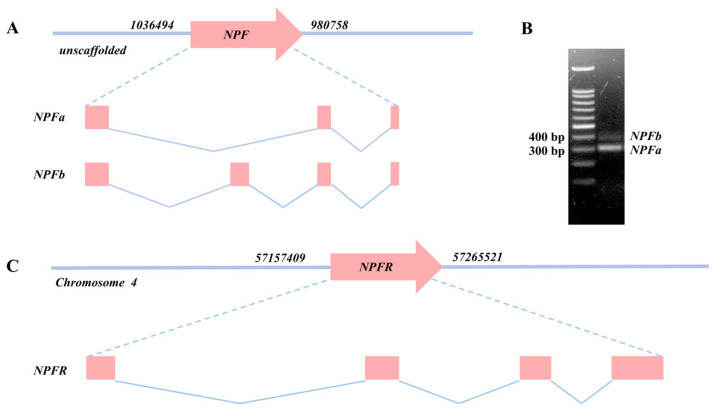
Gene structures of *NPF* and *NPFR* in *H. parallela*. The red boxes represents exons. (**A**) The *NPF* gene produces two transcript variants, *NPFa* and *NPFb*; *NPFa* lacks the second exon present in *NPFb*. (**B**) Validation of *NPFa* and *NPFb* transcript variants by RT-PCR using adult head cDNA. (**C**) Gene structure of the *NPFR* gene.

**Figure 2:**
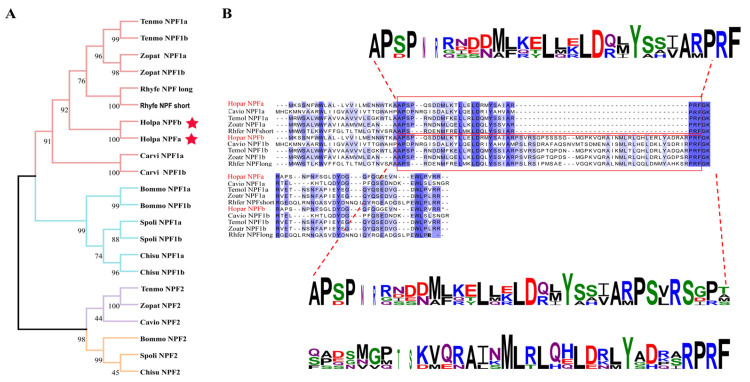
Phylogenetic analysis and multiple sequence alignment of insect NPF homologs. (**A**) A neighbor-joining phylogenetic tree of NPF homologs was constructed based on deduced amino acid sequences. Branches are color-coded by order and subtype: red for coleopteran NPF1 (NPF1a/1b) homologs, blue for lepidopteran NPF1 (NPF1a/1b) homologs, purple for coleopteran NPF2 homologs, and orange for lepidopteran NPF2 homologs. The NPF homologs from *H. parallela* are marked with red stars. Bootstrap values (1000 replicates) are indicated at the nodes. (**B**) Multiple sequence alignment of the conserved mature peptide regions. Sequences from *H. parallela* are highlighted in red, and conserved residues are shaded in blue. The mature bioactive peptide region is outlined by a red box and visualized as sequence logos, where amino acid height corresponds to conservation at each position. Species abbreviations are defined as follows: Tenmo, *Tenebrio molitor*; Zopat, *Zophobas atratus*; Rhyfe, *R. ferrugineus*; Holpa, *H. parallela*; Carvi, *Carabus violaceus*; Bommo, *Bombyx mori*; Spoli, *Spodoptera litura*; Chisu, *Chilo suppressalis*. Full references for all sequences are listed in the Supplementary Materials (Table S1).

**Figure 3:**
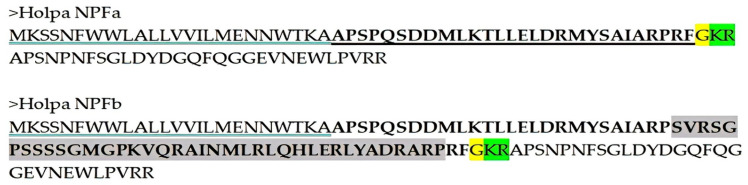
Prediction of mature NPF peptides from *H. parallela* revealed the sequence characteristics of two isoforms (NPFa and NPFb) of the NPF precursor protein. Signal peptides were underlined in green, and mature peptides were indicated in bold black. The amidation site (glycine residue, G) was highlighted in yellow, and dibasic cleavage sites (lysine residue, K; arginine residue, R) were marked in green. The mature peptide of NPFb exhibits a unique N-terminal extension sequence compared with that of NPFa, shown shaded.

In *H. parallela*, single copies of *NPF* and *NPFR* gene were identified. Two alternatively spliced transcript variants of *NPF*, designated *NPFa* and *NPFb*, encoding sequences of 255 and 369 bp, respectively, were found (Figure 1A). Both variants were validated by RT-PCR, cloning and sequencing using adult head cDNA as a PCR template (Figure S2). Notably, the mRNA expression level of *NPFa* was significantly higher than that of NPFb (Figure 1B). Sequence alignment revealed that *NPFa* lacks a 114 bp fragment corresponding to the second exon of *NPFb*. The mature peptides of NPFa and NPFb contained 28 and 66 amino acid residues, respectively, and shared a conserved RPRFamide motif at the C-terminus (Figure 2B). Signal peptide prediction identified a 24-residue hydrophobic N-terminal signal peptide in both NPFa and NPFb precursors, cleaved at Ala^24^/Ala^25^ (with an additional N-terminal extension sequence removed in NPFb). After cleavage at the conserved lysine–arginine (KR) site and glycine-mediated C-terminal amidation, the two isoforms produce mature peptides of 28 and 66 amino acids, respectively (Figure 3). Regarding *NPFR*, its ORF is 1188 bp in length and encodes a protein of 395 amino acids. Transmembrane domain prediction identified seven α-helical transmembrane domains, a characteristic feature of G protein-coupled receptors (Figure 1B).

**Figure 4:**
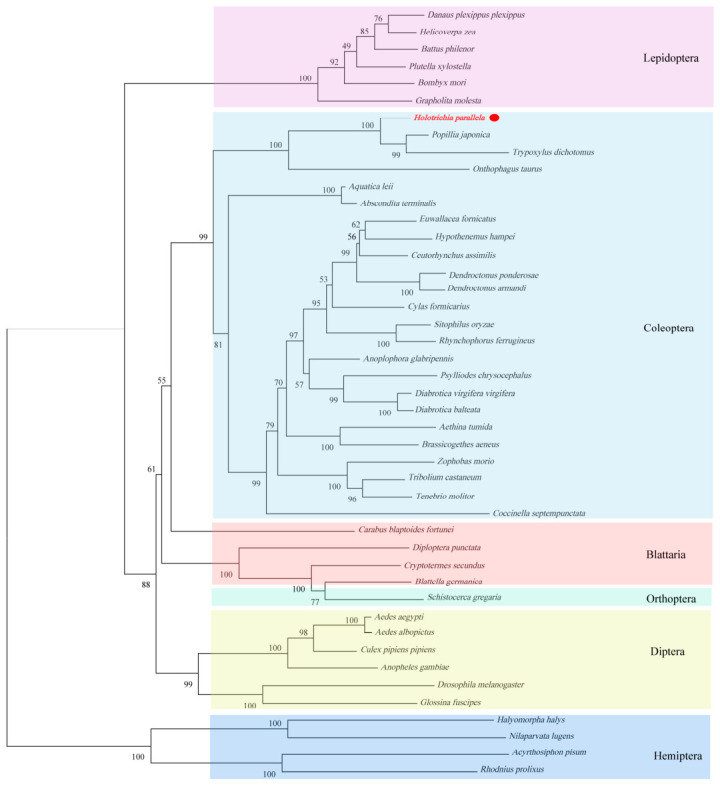
The phylogenetic tree, based on amino acid sequences of NPFR from *H. parallela* and other species, was constructed using the neighbor-joining method after ClustalW alignment, with a Poisson model and pairwise deletion of gaps were applied for tree construction. Bootstrap support values (%) are shown at each node. Branches are color-coded by insect order: purple for Lepidoptera, sky blue for Coleoptera, red for Blattaria, light green for Orthoptera, yellow for Diptera, and pale blue for Hemiptera. The red dot denotes the NPFR sequence of *H. parallela.* The accession numbers are provided in Supplementary Materials Table S4.

A phylogenetic tree of NPF precursors was constructed using protein sequences from Coleoptera and Lepidoptera. The analysis demonstrated that NPF1 and NPF2 formed two distinct clades, with *H. parallela* NPF clustering within the NPF1 monophyletic group (Figure 2A). Phylogenetic analysis of insect NPFRs revealed that NPFRs from the same order clustered into a separate clade (Figure 4). Furthermore, *H. parallela* NPFR exhibited the closest evolutionary relationship with NPFRs from other Scarabaeidae species, including *Popillia japonica*, *Trypoxylus dichotomus*, and *Onthophagus taurus*. Together, these findings support the evolutionary conservation of the insect *NPF* and *NPFR* gene families.

### 3.2. Expression Pattern Analysis of NPF and NPFR in Different Developmental Stages and Tissues

**Figure 5:**
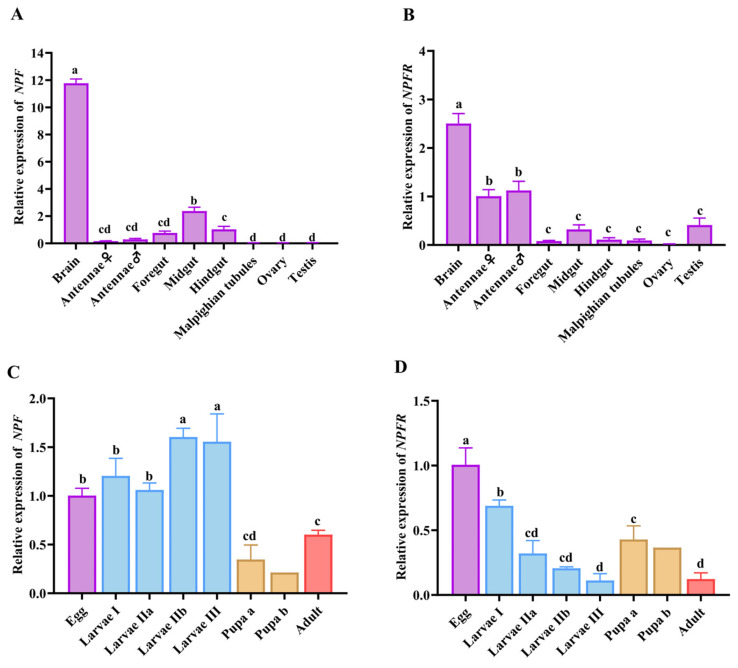
(**A**) Relative expression levels of NPF in various adult tissues. (**B**) Relative expression levels of NPFR in various adult tissues. (**C**) Relative expression levels of NPF across various developmental stages. (**D**) Relative expression levels of NPFR across various developmental stages. Expression profiles of *NPF* and *NPFR* in adult tissues and developmental stages of *H. parallela.* No significant sex-based differences in expression levels were observed in any tissue. Developmental stages included 3-day-old eggs; 10-day-old first-instar larvae (Larvae I); early (Larvae IIa) and late (Larvae IIb) second-instar larvae; 30-day-old third-instar larvae; early (Pupa a) and late (Pupa b) pupae, and 45-day-old adults; as detailed in Materials and Methods. Data were presented as means ± SE except for Pupa b (*n* = 2), which is shown as mean and was not included in statistical analysis. Different lowercase letters above error bars indicate statistically significant differences among stages or tissues (*P_adj_* < 0.05, one-way ANOVA followed by Tukey’s multiple comparisons test).

Using female adult brain tissue as the reference for relative expression normalization, we quantified *NPF* and *NPFR* transcript levels across multiple tissues of both sexes (brain, antennae, foregut, midgut, hindgut, Malpighian tubules, ovary, and testis) via RT-qPCR. Normalized expression data revealed that the two genes displayed distinct tissue-specific expression patterns (Figure 5A,B). For *NPF*, expression varied significantly across tissues (*p* < 0.0001), with the highest transcript levels detected in the brain, followed by the midgut, both significantly higher than in other tissues (*P_adj_* < 0.05). For *NPFR*, significant tissue-specific expression was also observed (*p* < 0.0001), peaking in the brain and followed by the antennae, with expression in these two tissues significantly higher than in all other tissues (*P_adj_* < 0.05). *NPFR* also showed moderate expression in the midgut and testis, though these levels were not significantly different from those in other examined tissues. Overall, no significant sex-related differences in gene expression were detected in any of the common tissues analyzed.

Using eggs as the reference stage, we further determined the relative expression of *NPF* and *NPFR* across developmental stages. Both genes were ubiquitously expressed across all life stages but displayed distinct temporal patterns (Figure 5C,D). Significant stage-specific variation was observed for *NPF* (*p* < 0.0001): transcripts reached the highest levels in late second- and third-instar larvae, followed by eggs and first-instar larvae, while expression in pupae was significantly reduced (*P_adj_* < 0.05). For *NPFR*, temporal expression also differed significantly across stages (*p* < 0.0001), peaking in eggs followed by first-instar larvae. Notably, *NPFR* expression in pupae was significantly higher than in third-instar larvae and 45-day-old adults. (*P*_adj_ = 0.0039 and 0.0051, respectively).

### 3.3. Effects of Stress Conditions on NPF and NPFR Transcript Level

**Figure 6:**
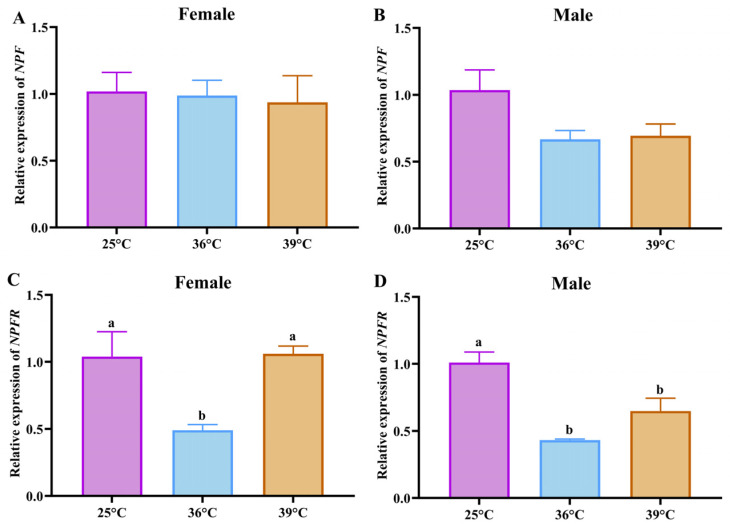
Effects of 3 h high-temperature stress on *NPF* and *NPFR* mRNA expression in the heads of female and male *H. parallela* adults. (**A**) Female *NPF*; (**B**) Male *NPF*; (**C**) Female *NPFR*; (**D**) Male *NPFR.* Transcript levels were normalized to *Actin* gene, with the 25 °C group used as the calibrator. Data were presented as means ± SE. Different lowercase letters above error bars indicate significant differences among temperature treatments within the same sex (*P*_adj_ < 0.05, one-way ANOVA followed by Tukey’s test).

Transcript levels of *NPF* and *NPFR* in adult heads exhibited distinct sex-specific responses to heat stress in *H. parallela.* In females, *NPF* expression showed no significant differences at 36 °C or 39 °C compared with the 25 °C control (*p* = 0.9315) (Figure 6A). In males, *NPF* levels decreased at 36 °C and slightly increased at 39 °C, although none of these changes reached statistical significance (*p* = 0.0689) (Figure 6B). For females, *NPFR* expression was significantly lower at 36 °C than at the 25 °C (*P*_adj_ = 0.0349) and at 39 °C (*P*_adj_ = 0.0299), and returned to baseline levels at 39 °C (*p* = 0.0211) (Figure 6C). In males, *NPFR* expression was significantly suppressed at both 36 °C and 39 °C relative to the 25 °C control (*P*_adj_ = 0.0007 and 0.0147, respectively) (Figure 6D).

**Figure 7:**
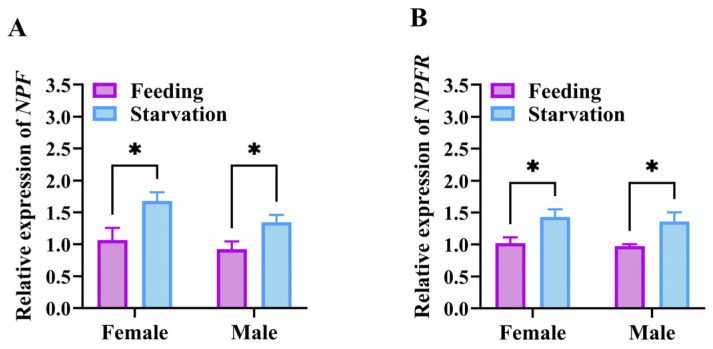
Effects of 96 h starvation on *NPF* and *NPFR* mRNA expression in the heads of *H. parallela* adults. (**A**) Relative *NPF* transcript levels in females and males; (**B**) Relative *NPFR* transcript levels in females and males. Transcript levels were normalized to the *Actin* gene. Data are presented as means ± SE. Asterisks indicate significant differences between fed control and starved groups within the same sex (*p* < 0.05, Student’s *t*-test).

In addition to heat stress, the expression patterns of *NPF* and *NPFR* under 96 h starvation stress were also examined in adult *H. parallela*. Transcript levels of *NPF* and *NPFR* were quantified in the heads of female and male individuals subjected to normal feeding or starvation stress. Both genes showed consistent expression patterns, with significant upregulation in starved individuals compared with fed controls, and no significant sex-based difference was detected. Female adults showed significantly higher *NPF* transcript abundance under starvation than under normal feeding (*p* = 0.0312), and a comparable induction pattern was detected in males (*p* = 0.0397), (Figure 7A). For *NPFR*, starvation stress led to a prominent increase in gene expression in females (*p* = 0.0363), and a similar significant upregulation was detected in starved males (*p* = 0.0489), (Figure 7B).

### 3.4. Determination of RNAi Efficiency

Double-stranded RNAs targeting *NPF* (dsNPF, 215 bp) and *NPFR* (dsNPFR, 457 bp) were synthesized in vitro, together with the control dsGFP (472 bp). Adults aged 20–40 days were selected for hemocoel injection, with 1 μL of dsRNA (2 μg) administered per individual. After injection, beetles were maintained on a diet of elm leaves. Silencing efficiency was evaluated by RT-qPCR at 48, 96, 144, and 192 h post-injection.

**Figure 8:**
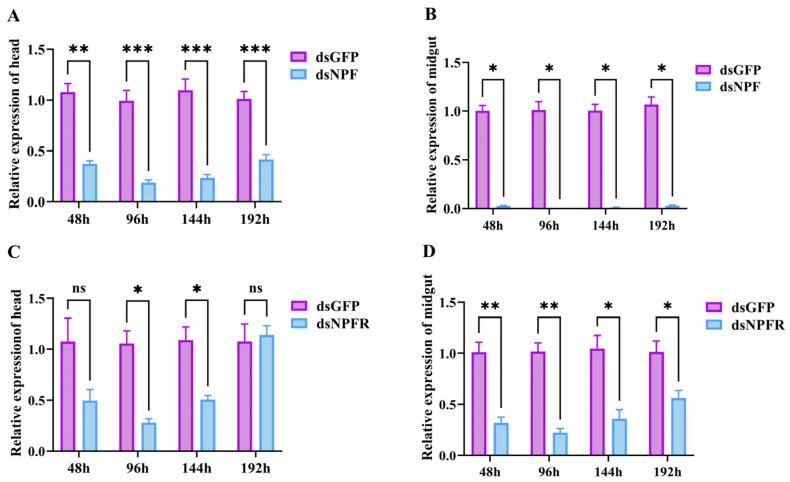
RNAi-mediated silencing of *NPF* (**A**,**B**) and *NPFR* (**C**,**D**) transcript levels in *H. parallela.* Relative mRNA expression levels were determined by RT-qPCR in insects injected with dsNPF, dsNPFR, or control dsGFP, and normalized to the dsGFP group. The *X*-axis represents hours post-injection (h). Data were presented as means ± SE. Statistical comparisons were performed using Student’s *t*-test (* *P*_adj_ < 0.05, ** *P*_adj_ < 0.01, *** *P*_adj_ < 0.001; ns, not significant).

Overall, the silencing efficiency of *NPF* was higher than that of *NPFR*, and both genes showed higher silencing efficiency in the midgut than in the head. Maximum silencing efficiency for both *NPF* and *NPFR* was achieved at 96 h post-injection: in the head, it was reduced by 81.05% for *NPF* (*P*_adj_ < 0.0001) and 73.50% for *NPFR* (*P*_adj_ = 0.0155) while in the midgut, it was reduced by 99.31% for *NPF* (*P*_adj_ = 0.0004) and 77.87% for *NPFR* (*P*_adj_ = 0.0032), respectively (Figure 8).

### 3.5. Effects of dsNPF and dsNPFR on Adult Food Consumption

**Figure 9:**
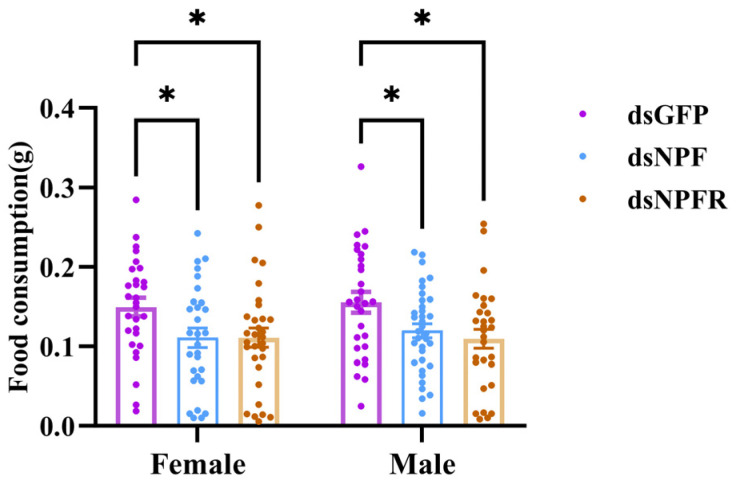
Effects of *NPF* and *NPFR* knockdown on food consumption (average daily intake on days 4 and 6 post-injection) in female and male *H. parallela* adults. Data are presented as mean ± SE. Asterisks indicate significant differences between the dsGFP control and dsNPF or dsNPFR treatment groups (*P*_adj_ < 0.05, one-way ANOVA followed by Dunnett’s test).

The average daily food consumption was calculated at 4 and 6 days post-injection. Knockdown of *NPF* or *NPFR* significantly reduced daily food intake in both female and male adults compared with the dsGFP control group (*p* = 0.0375 and 0.0142, respectively). In females, the mean daily food consumption was 0.11 ± 0.01 g and 0.12 ± 0.01 g in the dsNPF and dsNPFR groups, respectively, versus 0.15 ± 0.01 g in the control group (*P*_adj_ = 0.0491 and 0.0463, respectively). A similar pattern was observed in males, with values of 0.12 ± 0.01 g and 0.12 ± 0.01 g in the dsNPF and dsNPFR groups, respectively, relative to 0.16 ± 0.01 g in the control group (*P*_adj_ = 0.0438 and 0.0110, respectively). No significant differences were detected between the two dsRNA treatment groups. These results suggest that the NPF signaling system positively regulates food intake in *H. parallela* (Figure 9).

### 3.6. Effects of dsNPF and dsNPFR on Survival and Reproduction

**Figure 10:**
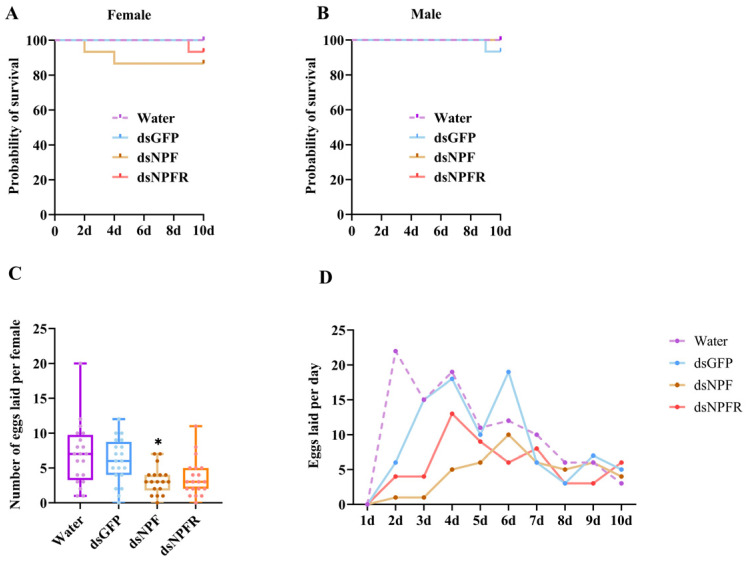
Effects of *NPF* and *NPFR* knockdown on survival and reproduction in *H. parallela* adults. (**A**,**B**) Survival curves of female and male adults. (**C**) Total egg production per female presented as boxplots, where each box indicates the median (central line), 25th (lower hinge) and 75th (upper hinge) percentiles; whiskers extend to 1.5× the interquartile range (IQR), and outliers are plotted as individual points. (**D**) Daily egg production per female. Data in (**D**) are presented as mean ± SE. Survival curves were analyzed using the log-rank test, with no significant differences detected among groups. Asterisks indicate significant differences in total egg production compared with the dsGFP control (*P*_adj_ < 0.05, one-way ANOVA with Dunnett’s multiple comparisons test). Survival and egg production were recorded daily for 10 consecutive days post-injection.

Survival analysis revealed that neither dsNPF nor dsNPFR injection caused significant changes in mortality in either sex compared with the water-injected and dsGFP-injected control groups (females: *p* = 0.2685; males: *p* = 0.3916) (Figure 10A,B). However, the total number of eggs laid per female over 10 days was significantly reduced in the dsNPF treatment group (*P*_adj_ = 0.0287), whereas a non-significant reduction was observed in the dsNPFR treatment group (*P*_adj_ = 0.0595) (Figure 10C). A similar trend was observed for daily egg production (Figure 10D). These results indicate that knockdown of *NPF* and its receptor gene does not affect short-term survival but suppresses reproduction in *H. parallela*.

### 3.7. Effects of dsNPF and dsNPFR on Energy Metabolism in Adults

**Figure 11:**
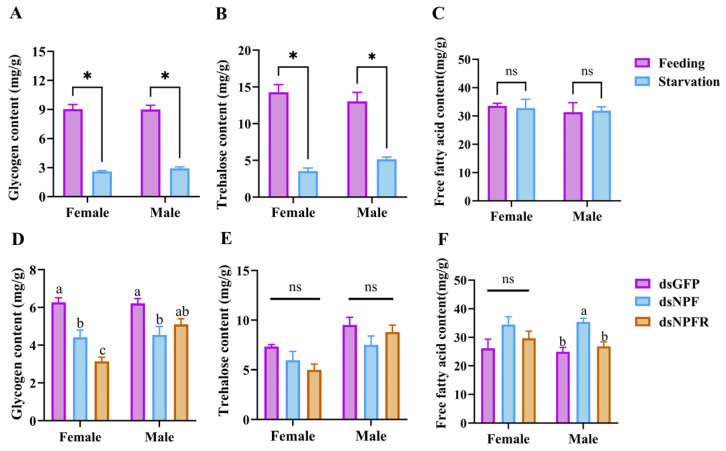
Effects of 4-day starvation (**A**–**C**) and *NPF* gene silencing (**D**–**F**) on energy metabolism in *H. parallela* adults. Data are presented as mean ± SE. In (**A**–**C**): ns, not significant, * *P*_adj_ < 0.05 (Student’s *t*-test). In (**D**–**F**): different lowercase letters indicate significant differences (*P*_adj_ < 0.05, one-way ANOVA followed by Dunnett’s test).

Compared with the continuously fed control group, 96 h starvation significantly reduced glycogen levels in both females and males (females: *P*_adj_ < 0.0001; males: *P*_adj_ < 0.0001) (Figure 11A). Trehalose levels were also significantly decreased in both females and males under starvation (females: *P*_adj_ = 0.0002; males: *P*_adj_ = 0.0016) (Figure 11B), whereas FFA content showed no significant difference between the two groups in either sex (females: *P*_adj_ = 0.8172; *P*_adj_ = 0.8906) (Figure 11C).

In RNAi experiments, compared with the dsGFP control group, glycogen content was significantly decreased in females treated with dsNPF and dsNPFR (*P*_adj_ = 0.0027 and <0.0001, respectively), whereas in males, only dsNPF treatment caused a significant reduction (*P*_adj_ = 0.0131) (Figure 11D). Trehalose levels showed no statistically significant difference among treatments in either sex (females: *p* = 0.0701; Males: *p* = 0.2446) (Figure 11E). For FFA content, males treated with dsNPF exhibited a significant increase relative to the dsGFP control group (*P*_adj_ = 0.0012), while no significant changes were observed in females under any RNAi treatment group (*p* = 0.1742) (Figure 11F).

These findings indicated that the NPF/NPFR signaling pathway regulates energy metabolism in *H. parallela* adults, particularly by promoting glycogen storage or synthesis.

## 4. Discussion

*Holotrichia parallela* is a significant soil-dwelling pest that damages the root systems of various crops, and its cryptic subterranean life history substantially increases the difficulty of field monitoring and control [46,47]. Chemical control readily leads to insecticide resistance and ecological risks [48], while biological control methods are often limited by practical constraints in large-scale application. Consequently, there is an urgent need to identify novel molecular targets. As a multifunctional neuropeptide, the NPF signaling system is extensively involved in regulating a range of core physiological processes in insects, including feeding, metabolism, reproduction, and behavioral rhythms, rendering it a promising molecular target for pest control [6]. In this study, we cloned the full-length coding sequences of *NPF* and *NPFR*, characterized their expression profiles, and performed RNAi-based functional analyses. Our results demonstrate that these genes regulate feeding behavior and energy metabolism in *H. parallela*, providing a foundation for the development of novel, environmentally friendly control strategies. In most invertebrates, the *NPF* gene is present as a single copy per haploid genome. However, exceptions exist in a few insect groups; lepidopterans frequently possess two *NPF* homologs, and *NPF1* often generates alternative splicing variants [38,49,50]. In Coleoptera, two *NPF* genes and alternative splicing of *NPF1* have also been reported in *Carabus violaceus* and *Tenebrio molitor* [40,51]. In this study, only one *NPF* gene was identified from *H. parallela*, which is a homolog of *NPF1*, consistent with findings in *R. ferrugineus* and *D. armandi* [16,17]. Furthermore, multiple sequence alignment revealed that coleopteran NPFs are less conserved than their lepidopteran counterparts [52], suggesting that the NPF system in Coleoptera has undergone more rapid divergence or adaptive evolution, which may be related to their omnivorous feeding habits and survival strategies.

Alternative splicing, a conserved post-transcriptional regulatory mechanism, serves as a significant molecular process for generating functional diversity of neuropeptides [51]. In this investigation, we identified that the *NPF* gene in *H. parallela* produces two alternatively spliced isoforms, *NPFa* and *NPFb*, through exon skipping. Both isoforms contain a conserved C-terminal RPRFamide motif and an amidation site. However, they exhibit notable structural differences: the mature peptide of NPFb possesses a 38-amino-acid N-terminal extension absent in NPFa. Research in *Drosophila melanogaster* and *B. mori* has indicated that N-terminal extensions can influence receptor binding affinity, signal transduction efficiency, or proteolytic stability of neuropeptides in vivo [7]. In the present study, the mRNA expression level of NPFa in adult heads was substantially higher than that of NPFb, suggesting that NPFa may be the primary effector of NPF signaling during the adult stage. It is important to note that whether both isoforms are produced and released as functional mature peptides by neurosecretory cells, or whether they possess distinct physiological functions (e.g., in feeding, reproduction, or stress responses), remains unclear. Future studies should compare their abilities to activate NPFR using calcium mobilization assays with in vitro synthesized mature peptides and elucidate their functional divergence in the regulation of feeding and other physiological processes through in vivo injection experiments.

Through spatiotemporal expression profiling, we found that *NPF* in *H. parallela* was most highly expressed in the brain, consistent with reports that *NPF* is localized in the central nervous system, particularly in neuroendocrine centers such as the interbrain [8,53,54]. *NPF* was also highly expressed in the midgut, supporting previous reports that *NPF* is involved in the regulation of feeding and digestion, as observed in the brown planthopper and cotton bollworm [23,55]. Regarding *NPFR*, previous studies have demonstrated its high expression in the central nervous system, intestine [17], and peripheral nervous system [56]. In this study, we observed high *NPFR* mRNA expression in the antennae. Given the central role of antennae in insect olfactory perception, this expression pattern suggests that NPF-NPFR signaling may participate in olfactory regulation. Recent studies have also shown that *NPF* regulates the expression of odorant-binding protein OBP5 to influence feeding in the brown planthopper [57]. Temporally, both *NPF* and *NPFR* were transcribed throughout all developmental stages of *H. parallela*, indicating that they play important roles across the entire life cycle. *NPF* expression peaked in second- and third-instar larvae, consistent with its functions in feeding behavior and energy homeostasis [55], which aligns with reports in the cotton bollworm [23]. In contrast, *NPFR* expression peaked in eggs and early first-instar larvae, suggesting a role in early development, possibly in cell proliferation and differentiation [7,58].

Short-term high-temperature stress did not significantly alter the transcriptional level of *NPF* in *H. parallela*; however, its receptor gene responded to heat stress with sexually dimorphic expression patterns. The heat-induced suppression of *NPFR* expression in females was restored as the temperature increased, whereas the recovery ability in males was significantly weaker than that in females. These findings indicate that the NPF–NPFR signaling pathway participates in the short-term thermal stress response of *H. parallela* in a sex-specific manner, and that *NPFR* is the core functional gene through which this pathway senses high-temperature stress. The differential expression responses between sexes also suggest that males and females possess different thermal adaptation strategies. In contrast, *NPFR* in aphids may not directly participate in the response to high-temperature stress [33]. This difference reflects species-specific thermal adaptation strategies: different species may exhibit varying degrees of dependence on neuropeptide signaling for thermal stress responses. *H. parallela* may utilize neuropeptide signaling for sex-dependent thermal responses, whereas aphids may rely primarily on other stress pathways (e.g., heat shock proteins and cathepsin B) [59] rather than NPF–NPFR-mediated pathways.

The role of neuropeptide F (*NPF*) in regulating feeding behavior has been confirmed in multiple insect species; however, its specific behavioral effects are species-specific, either promoting or inhibiting food intake or foraging activity [54]. In *H. parallela*, starvation significantly upregulated the transcript levels of both *NPF* and its receptor (*NPFR*), and knockdown of either gene significantly reduced food intake in both male and female adults, confirming that the NPF signaling system positively regulates feeding behavior in this species. These results are consistent with reports in *D. melanogaster* [21], *Schistocerca gregaria* [60], *Acyrthosiphon pisum* [61], *D. armandi* [17], and *Helicoverpa armigera* [24].

Furthermore, knockdown of NPF or NPFR reduced glycogen content in *H. parallela* adults and led to the accumulation of free fatty acids in males, which is consistent with effects reported in other insect species [17,54,62], further supporting the role of NPF signaling in regulating carbohydrate and lipid metabolism. It should be noted that a causal relationship exists between feeding inhibition and glycogen reduction: NPF/NPFR silencing resulted in a significant decrease in food intake, and reduced intake inevitably leads to insufficient exogenous carbohydrate supply, which may be the most direct cause of decreased glycogen content. However, reduced food intake alone cannot fully account for all observed metabolic changes. First, the magnitude of glycogen reduction was not completely related to the magnitude of feeding reduction in *H. parallela* (Figure 11D), suggesting that NPF signaling may also directly regulate glycogen synthesis or degradation pathways. Second, the accumulation of free fatty acids in males following *NPF* silencing, while reduced food intake is typically accompanied by increased fat mobilization, may represent a compensatory energy response. Moreover, this phenomenon occurred only in males, suggesting that the regulation of lipid metabolism by NPF signaling may exhibit sexual dimorphism. Additionally, in mammals and *Drosophila*, NPY/NPF signaling has been shown to regulate glycogen metabolism via glycogen synthase kinase-3β (GSK-3β) [62]. Therefore, we hypothesize that NPF signaling in *H. parallela* regulates energy metabolism through a dual mechanism: indirectly by modulating feeding behavior to affect substrate supply, and directly by acting on metabolic tissues (e.g., fat body and intestine) to regulate glycogen synthesis and degradation. Our RNAi-based transcriptomic analysis also supports this view, showing significant enrichment of differentially expressed genes related to glucose and lipid metabolism following NPF silencing.

In insects, the homeostatic regulation of energy metabolism involves the coordinated action of multiple signaling pathways, among which the insulin/insulin-like growth factor signaling (IIS) pathway and the target of rapamycin (TOR) pathway are central regulatory hubs. In this study, the phenotypes observed following NPF/NPFR silencing—reduced glycogen content and decreased feeding—partially resemble those resulting from insulin signaling deficiency. In *Drosophila*, functional crosstalk between *NPF* and insulin signaling has been demonstrated. Midgut-derived *NPF* functions as a glucose-responsive incretin that promotes the secretion of insulin-like peptides from insulin-producing cells (IPCs), thereby regulating lipid metabolism while suppressing the production of adipokinetic hormone (AKH) [62]. This finding established the existence of a “gut–insular axis” in invertebrates for the first time. Although NPF in *H. parallela* is highly expressed in both the brain and midgut, suggesting the possible existence of a similar gut–brain endocrine axis, whether NPF signaling influences glycogen metabolism by modulating the balance between insulin-like peptides (ILPs) and AKH requires experimental verification. Furthermore, a recent study in *Ostrinia furnacalis* demonstrated that NPFR binds to Gα proteins to activate the second messengers cAMP and Ca^2+^, which in turn phosphorylate AMP-activated protein kinase (AMPK), thereby regulating lipid and glycogen synthesis and metabolism [63]. This finding reveals an NPFR–AMPK signal transduction pathway within the NPF signaling system. In mammals, AMPK is known as a “cellular energy sensor” that is activated under energy-deficient conditions to promote catabolism and inhibit anabolism. Based on the above studies, we speculate that NPF signaling in *H. parallela* may regulate energy metabolism through the following two mechanisms: functional crosstalk with the IIS/AKH pathway to influence metabolic homeostasis; or, direct regulation of glycogen and lipid metabolism via activation of the AMPK signaling pathway downstream of its receptor. The upregulation of NPF and NPFR expression under starvation stress (Figure 7) may represent a compensatory response aimed at maintaining homeostasis by promoting feeding and activating AMPK to mobilize energy reserves.

In contrast to the elevated trehalose levels observed in *Plutella xylostella* [52], *D. armandi* [17], and *H. armigera* [23], trehalose levels in *H. parallela* did not change significantly following NPF/NPFR knockdown. Time-series data from armyworms suggest that the effect of NPF signaling on trehalose may represent a transient compensatory response rather than a sustained change [64]. Another possible explanation involves species-specific differences in energy substrate mobilization strategies. Starvation stress experiments further revealed that under short-term nutrient deprivation, *H. parallela* preferentially mobilizes glycogen and trehalose to meet energy demands, resulting in a significant decrease in both substrates. Although silencing the NPF pathway alone caused a moderate decrease in glycogen content, it did not recapitulate the complete energy depletion phenotype observed under starvation conditions. This suggests that NPF signaling may not be the sole factor mediating the starvation response.

Regarding reproductive regulation, *NPF* silencing in *H. parallela* resulted in a significant decrease in the oviposition rate of females, indicating that this gene plays a key role in reproduction. This observation is consistent with previous reports in *H. armigera* [24] and *O. furnacalis* [65]. Considering the results of energy metabolism assays, the reduction in fecundity may be a combined effect of *NPF* silencing: feeding inhibition leads to insufficient nutrient intake, while disruption of glycogen metabolism results in an inadequate energy supply. Consequently, the energy investment required for vitellogenesis and oviposition may be limited. NPF signaling regulates insect fecundity by coupling nutritional metabolism with reproductive energy supply [66], a regulatory mechanism that appears to be conserved across insects [60]. In contrast, *NPFR* knockdown did not produce a significant inhibitory effect on fecundity. However, this lack of significance may be attributable to the relatively low knockdown efficiency and shorter effective duration of *NPFR* silencing, rather than indicating that this receptor plays no role in reproductive regulation. Importantly, the reduction in fecundity was not secondary to decreased adult survival, as *NPF* silencing did not significantly affect short-term adult survival in *H. parallela*. This finding differs from previous reports in the red palm weevil and several lepidopteran species, in which silencing of *NPF* and its receptor genes resulted in increased mortality [17,24].

In summary, this study represents the first cloning and characterization of *NPF* and *NPFR* genes in the scarabaeid pest *H. parallela* (Coleoptera) and reveals the existence of two alternatively spliced isoforms of *NPF* with markedly different expression levels. Furthermore, this study provides critical molecular and functional data for understanding the physiological functions of NPF signaling in scarabaeid beetles, establishing the central role of this signaling system in regulating feeding, energy metabolism, and reproduction. Although the RNAi injection method is not directly applicable in the field, these findings provide a theoretical basis and target validation for the future development of behavior-based control strategies targeting the NPF signaling pathway, such as the delivery of dsRNA via transgenic plants or nanomaterials.

## 5. Conclusions

In summary, in *H. parallela* adults, *NPF* and *NPFR* are predominantly localized in the brain and midgut, while *NPFR* is also abundantly expressed in the antennae. NPF–NPFR signaling is involved in regulating feeding behavior, energy metabolism, and reproduction adults. These findings advance our understanding of the physiological functions of this signaling system in coleopteran insects and provide a foundation for developing novel, eco-friendly approaches to manage *H. parallela*. However, the precise molecular mechanisms by which the NPF–NPFR signaling system regulates feeding, energy metabolism, and reproduction remain to be fully elucidated and require further investigation.

## Data Availability

The data presented in this study are available in the article.

## Supplementary Materials

The following supporting information can be downloaded at: https://www.mdpi.com/article/10.3390/biology15120903/s1, Table S1. Information on neuropeptide F (NPF) homolog sequences used for phylogenetic analysis and multiple sequence alignment; Table S2. Primer sequences used in the research; Table S3. Primers for dsRNA synthesis; Table S4. Species and GenBank accession numbers of NPFR homologs shown in the phylogenetic tree; Table S5. Stability evaluation indicators of *Actin* reference gene; Table S6. Detailed statistical results for all experiments. Figure S1. Ct values of *Actin* reference gene under heat and starvation stress in *Holotrichia parallela*. In each box, the lower quartile (25th percentile) and upper quartile (75th percentile) are shown. The whiskers represent the minimum and maximum values of the dataset. The horizontal line inside each box indicates the median value; Figure S2. Validation of NPFa and NPFb transcript variants by RT-PCR using adult head cDNA.
