# Supplementary material for: Identification of Neuropeptide F (NPF) Signaling and Associated Regulation of Food Intake in the Dark Black Chafer Beetle Holotrichia parallela
Source: Biology (Basel). 2026 Jun 9;15(12):903. doi: 10.3390/biology15120903 (PMC13295887; doi:10.3390/biology15120903)
Supplement: Supplementary file 1 [file biology-15-00903-s001.zip › biology-4315134-supplementary.pdf]

Supplementary Information

Table S1. Information on neuropeptide F (NPF) homolog sequences used for phylogenetic analysis and multiple sequence alignment.

Abbreviation	Species name	Gene/Peptide	GenBank Accession No.	Source
Holpa	Holotrichia parallela	NPFa	-	Present study
Holpa	Holotrichia parallela	NPFb	-	Present study
Carvi	Carabus violaceus	NPF1a	-	[17]
Carvi	Carabus violaceus	NPF1b	-	[17]
Carvi	Carabus violaceus	NPF2	-	[17]
Tenmo	Tenebrio molitor	NPF1a	ON110516	-
Tenmo	Tenebrio molitor	NPF1b	ON110517	-
Tenmo	Tenebrio molitor	NPF2	ON110518	-
Zopat	Zophobas atratus	NPF1a	ON155949	-
Zopat	Zophobas atratus	NPF1b	ON155950	-
Zopat	Zophobas atratus	NPF2	ON155951	-
Rhyfe	Rhynchophorus ferrugineus	NPFshort	QGA72566.1	-
Rhyfe	Rhynchophorus ferrugineus	NPFlong	-	[38]
Spoli	Spodoptera litura	NPF1a	XP_022825973	-
Spoli	Spodoptera litura	NPF1b	XP_022825972	-
Spoli	Spodoptera litura	NPF2	XP_022834589	-
Chisu	Chilo suppressalis	NPF1a	ALM30332.1	-
Chisu	Chilo suppressalis	NPF1b	ALM30333.1	-
Chisu	Chilo suppressalis	NPF2	ALM30334.1	-
Bommo	Bombyx mori	NPF1a	NP_001124355.1	-
Bommo	Bombyx mori	NPF1b	NP_001166883.1	-
Bommo	Bombyx mori	NPF2	NP_001124361.1	-

Note: GenBank accession numbers are provided where available; a dash (–) indicates sequences without public database entries. The novel *H. parallela* NPFa, NPFb, and NPFR sequences identified in this study also lack public GenBank accessions. All references cited in this table are listed in the main reference section of the manuscript. Full-length amino acid sequences for all entries without GenBank accessions (including *H. parallela* NPFa, NPFb, and NPFR) are provided in the supplementary materials.

>Holpa NPFa

MKSSNFWWLALLVVILMENNWTKAAPSPQSDDMLKTLLELDRMYSAIARPRFGKRAPSNPNFSGLDYDGQFQGGEVNEWLPVRR

> Holpa NPFb

MKSSNFWWLALLVVILMENNWTKAAPSPQSDDMLKTLLELDRMYSAIARPSVRSGPSSSSGMGPKVQRAINMLRLQHLERLYADRARPR
FGKRAPSNPNFSGLDYDGQFQGGEVNEWLPVRR*

> Holpa NPFR

MEIHINSSTVIDIINKSNEVDPDLISSYMQNRAVNEPAYSILILMYSLLILTGASGNILVIVSVVRKPAMRTPRNMFIVNLAVSDLLLCTVTMPL
TLMEILTKYWPLGNYLILCKMKSALQATSIFVTTITIAAIALDRYRVIVYPTRESLNLCLTGAILLAIWIMASILASPLFIYTQLVHHDLKINGT
DLGINFCIEDWPFKDGRALYSIFSIIFQYLVPIIIVSSAYLSIYLKLRYRFASGFVSNEEFSQNSTRRQTRGRKLKRTNMLLISIALVFCISWLPLNL
FNLIADIYSSEEFSQKTGIVIYAVCHMMGMSSACSNPVLYGWLNENFWKEFKDIMCLSTSENNAGAKRSSIKGTSRKTLGKGTPDVVVMA
GEFHAGNMSTEMTNLTS*

>Carvi Neuropeptide F1 transcript a

MHCKMNVAARWLIIGVAVVIVTTGWAHPAPDPNRGISDALKYLQELDRIYAHVAMPRFGKRTELKHTLQDYDGPFQSEDNDKEWLSLS
NGR

>Carvi Neuropeptide F1 transcript b

MHCKMNVAARWLIIGVAVVIVTTGWAHPAPDPNRGISDALKYLQELDRIYAHVAMPSLRSDRAFAQSNVMTSDMENAISMLRLHELDK
LYSDRIRPRFGKRTELKHTLQDYDGPFQSEDNDKEWLSLSNGR

>Carvi Neuropeptide F2 (NPY)

MFNKHMLVVCTVSLVLVLMLDGTRSHPVQPKRPQKFENEEQLRQYIREVKQYLDETVRTGRYGKRNYMAPSMIFKSYPKQDEPEVNNDY
DYYAQFNI

>Rhyfe neuropeptide F-long

MWWSTLKWVFFGLTLTMLGTNVSRAAPSRDENMFRELMKLDQLYSSIARPSLRSIPMSAEVGQKVQRALNMLRLQQLDNMYAHKSRPR
FGKRGEGQLRNNGASVDYDNNQIQYRGEADGSLPEWLPLRR

Table S2. Primers for dsRNA synthesis

Usage	Primer name	Primer sequence(5′-3′)
NPF (+)	T7-NPF-F	taatacgactcactatagggAGAGACTGTACGCCGATAGAG
	NPF-R	GGCTTCTCTGCATGAACGAT
NPF (-)	NPF-F	AGAGACTGTACGCCGATAGAG
	T7-NPF-R	taatacgactcactatagggGGCTTCTCTGCATGAACGAT
NPFR (+)	T7-NPFR-F	taatacgactcactatagggGTCAGAAAACCGGCATTGTT
	NPFR-R	AATTCTCCAGCCATGACGAC
NPFR (-)	NPFR-F	GTCAGAAAACCGGCATTGTT
	T7-NPFR-R	taatacgactcactatagggAATTCTCCAGCCATGACGAC
GFP(+)	T7-GFP-F	taatacgactcactatagggCACAAGTTCAGCGTGTCCG
	GFP409-R	TgCCgTTCTTCTgCTTgTCg
GFP(-)	GFP409-F	CACAAgTTCAgCgTgTCCg
	T7-GFP-R	taatacgactcactatagggTGCCGTTCTTCTGCTTGTCG

Table S3. Primer sequences used in this study.

Primer	Primer sequence	Usage
NPF-F	CGTTATCTCATCTTCGGACT	clone
NPF-R	GTGAACTGGCTCAATACCTA	clone
NPF-F	AGGCTTCTCTGCATGAACGAT	clone
NPF-R	TCCACAGAGCGACGATATGC	clone
NPFR-F	AGCTCGTCAGATTAGTCATT	clone
NPFR-R	GATCGTATATCCGACAAGAGA	clone
NPF-F	CGTTATCTCATCTTCGGACT	clone
5′adaptor	GCTGTCAACGATACGCTACGTAACGGCATGACAGTGGGIIGGGIIGGGIG	RACE
5.3′outer	GCTGTCAACGATACGCTACGTAAC	RACE
5.3′inner	GCTACGTAACGGCATGACAGTG	RACE
RC1066-FT1	AATTATTGTTTCGAGTGCATATCTGAGT	RACE
RC1066-FT2	GCTTCGTCAGCAATGAAGAGTTTAG	RACE
RC1066-RT1	ATGTCTGCTATGAGGTTGAAGAGGT	RACE
qHpNPF-F	TTCTGGTGGCTGGCATTACT	RT-qPCR
qHpNPF-R	TCGGTCTTGCTATGGCTGAAT	RT-qPCR
qHpNPFR-F	CGGTGTTCCCTTGCCCAATGT	RT-qPCR
qHpNPFR-R	TGCTATACGGCTGGTTGAACGA	RT-qPCR
actin-F*	ATGTTGCCATCCAAGCTGTA	RT-qPCR
actin-R*	CCAAACGCAAAATAGCATGA	RT-qPCR
GAPDH-F**	TGCATGCTATCACAGCTACGC	RT-qPCR
GAPDH-R**	AATACCTTTTAGTGGTCCTTCCG	RT-qPCR

Note: * Actin primers were designed based on the *H. parallela* actin sequence (GenBank Accession No. MT991084) reported in reference [44]. ** GAPDH primers were designed based on the *H. parallela* GAPDH sequence reported in reference [45], for which no public GenBank accession number was available. All other primers were designed in this study based on the NPF and NPFR sequences identified in the present work.

Table S4. .Species and GenBank accession numbers of NPFR homologs shown in the phylogenetic tree.

NO.	Species	GenBank accession No.
1	*Abscondita terminalis*	KAF5296736.1
2	*Acyrthosiphon pisum*	XP 029342450.1
3	*Aedes aegypti*	AGX85007.1
4	*Aedes albopictus*	KXJ70537.1
5	*Aethina tumida*	XP 019877913.1
6	*Anopheles gambiae*	AAT81602.1
7	*Anoplophora glabripennis*	XP 018564523.1
8	*Aquatica leii*	KAK4875548.1
9	*Battus philenor*	XP 068625534.1
10	*Blattella germanica*	PSN42328.1
11	*Bombyx mori*	NP 001127739.1
12	*Brassicogethes aeneus*	CAH0555734.1
13	*Carabus blaptoides fortunei*	GLV32679.1
14	*Ceutorhynchus assimilis*	CAH1122359.1
15	*Coccinella septempunctata*	XP 044754601.1
16	*Cryptotermes secundus*	XP 023707897.1
17	*Culex pipiens pipiens*	KAL1399351.1
18	*Cylas formicarius*	XP 060529392.
19	*Danaus plexippus plexippus*	OWR47452.1
20	*Dendroctonus ponderosae*	XP019756681.1
21	*Dendroctonus armandi*	QXU63634.1
22	*Diabrotica balteata*	CAG9838277.1
23	*Diabrotica virgifera virgifera*	XP 050509506.1
24	*Diploptera punctata*	KAJ9598827.1
25	*Drosophila melanogaster*	AAK50050.1
26	*Euwallacea fornicatus*	XP 066146133.1
27	*Glossina fuscipes*	XP 037884831.1
28	*Grapholita molesta*	QPZ46777.1
29	*Halyomorpha halys*	XP 014291375.3
30	*Helicoverpa zea*	XP 047036011.1
**31**	***Holotrichia parallela***	
32	*Hypothenemus hampei*	KAL1494616.1
33	*Nilaparvata lugens*	XP 039300489.1
34	*Onthophagus taurus*	XP 022918568.1
35	*Plutella xylostella*	XP 011565078.1
36	*Popillia japonica*	KAK9731276.1 7
37	*Psylliodes chrysocephalus*	CAH1111132.1
38	*Rhodnius prolixus*	AKO62910.1
39	*Rhynchophorus ferrugineus*	QGA72501.1
40	*Schistocerca gregaria*	XP 049862343.1
41	*Sitophilus oryzae*	XP 030754183.1
42	*Tenebrio molitor*	KAJ3626067.1
43	*Tribolium castaneum*	XP 008198438.1
44	*Trypoxylus dichotomus*	GJQ85491.1
45	*Zophobas morio*	XP 063925634.1

Table S5 Stability evaluation indicators of *Actin* reference gene

Heat stress assay	Female			Male		
	25℃	36℃	39℃	25℃	36℃	39℃
Intra-group coefficient of variation (%)	2.216	2.003	2.675	2.866	2.929	2.581
Overall coefficient of variation (%)			2.128			2.644
Overall standard deviation			0.357			0.436
One-way ANOVA (P value)			0.865			0.668
Relative expression level of genes	1.000	1.009	1.005	1.000	0.984	0.999

Starvation assay	Female		Male	
	Feeding	Starvation	Feeding	Starvation
Intra-group coefficient of variation (%)	1.471	0.838	1.889	1.953
Overall coefficient of variation (%)		1.137		2.192
Overall standard deviation		0.195		0.372
Student's t-test (P value)		0.75		0.9
Relative expression level of genes	1.000	0.995	1.000	1.024

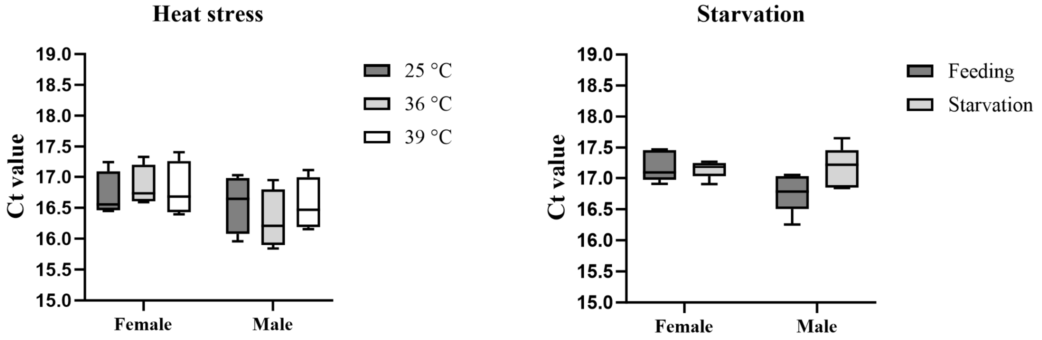

Figure S1 Ct values of *Actin* reference gene under heat and starvation stress in *Holotrichia parallela*. In each box, the lower quartile (25th percentile)and upper quartile (75th percentile) are shown. The whiskers represent the minimum and maximum values of the dataset. The horizontal line inside each box indicates the median value.

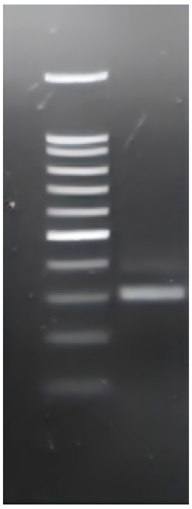

Figure S2 Validation of NPFa and NPFb transcript variants by RT-PCR using adult head cDNA.

Table S6. Detailed statistical results for all experiments.

**Experiment**	**Figure**	**Group**	**Statistics**
Spatiotemporal profile	Figure. 5A	Tissue-profile (*NPF*)	Test: One-way ANOVA + Tukey's test<br>*F* = 543.6, df = 8, 20, *P* < 0.0001 Sig.: ***<br>Adjusted *P* values for multiple comparisons are as follows:<br>Brain vs. Antennae♀: <0.0001, ****<br>Brain vs. Antennae♂: <0.0001, ****<br>Brain vs. Foregut: <0.0001, ****<br>Brain vs. Midgut: <0.0001, ****<br>Brain vs. Hindgut: <0.0001, ****<br>Brain vs. Malpighian tubules: <0.0001, ****<br>Brain vs. Ovary: <0.0001, ****<br>Brain vs. Testis: <0.0001, ****<br>Antennae♀ vs. Antennae♂: 0.9997, ns<br>Antennae♀ vs. Foregut: 0.2993, ns<br>Antennae♀ vs. Midgut: <0.0001, ****<br>Antennae♀ vs. Hindgut: 0.0413, *<br>Antennae♀ vs. Malpighian tubules: 0.9998, ns<br>Antennae♀ vs. Ovary: 0.9997, ns<br>Antennae♀ vs. Testis: 0.9998, ns<br>Antennae♂ vs. Foregut: 0.6748, ns<br>Antennae♂ vs. Midgut: <0.0001, ****<br>Antennae♂ vs. Hindgut: 0.1699, ns<br>Antennae♂ vs. Malpighian tubules: 0.9818, ns<br>Antennae♂ vs. Ovary: 0.9806, ns<br>Antennae♂ vs. Testis: 0.984, ns<br>Foregut vs. Midgut: 0.0002, ***<br>Foregut vs. Hindgut: 0.9805, ns<br>Foregut vs. Malpighian tubules: 0.1735, ns<br>Foregut vs. Ovary: 0.1703, ns<br>Foregut vs. Testis: 0.1797, ns<br>Midgut vs. Hindgut: 0.0014, **<br>Midgut vs. Malpighian tubules: <0.0001, ****<br>Midgut vs. Ovary: <0.0001, ****<br>Midgut vs. Testis: <0.0001, ****<br>Hindgut vs. Malpighian tubules: 0.0239, *<br>Hindgut vs. Ovary: 0.0233, *<br>Hindgut vs. Testis: 0.0249, *<br>Malpighian tubules vs. Ovary: >0.9999, ns<br>Malpighian tubules vs. Testis: >0.9999, ns<br>Ovary vs. Testis: >0.9999, ns
	Figure. 5B	Tissue-profile (*NPFR*)	Test: One-way ANOVA + Tukey's test<br>*F* =42.14, df = 8, 20, *P* < 0.0001 Sig.: ***<br>Adjusted *P* values for multiple comparisons are as follows:<br>Brain vs. Antennae♀: <0.0001, ****<br>Brain vs. Antennae♂: <0.0001, ****

Experiment	Figure	Group	Statistics
			Brain vs. Foregut: <0.0001, ****<br>Brain vs. Midgut: <0.0001, ****<br>Brain vs. Hindgut: <0.0001, ****<br>Brain vs. Malpighian tubules: <0.0001, ****<br>Brain vs. Ovary: <0.0001, ****<br>Brain vs. Testis: <0.0001, ****<br>Antennae♀ vs. Antennae♂: 0.9988, ns<br>Antennae♀ vs. Foregut: 0.0016, **<br>Antennae♀ vs. Midgut: 0.0276, *<br>Antennae♀ vs. Hindgut: 0.0023, **<br>Antennae♀ vs. Malpighian tubules: 0.0018, **<br>Antennae♀ vs. Ovary: 0.0008, ***<br>Antennae♀ vs. Testis: 0.075, ns<br>Antennae♂ vs. Foregut: 0.0008, ***<br>Antennae♂ vs. Midgut: 0.0124, *<br>Antennae♂ vs. Hindgut: 0.0012, **<br>Antennae♂ vs. Malpighian tubules: 0.001, ***<br>Antennae♂ vs. Ovary: 0.0004, ***<br>Antennae♂ vs. Testis: 0.0328, *<br>Foregut vs. Midgut: 0.9406, ns<br>Foregut vs. Hindgut: >0.9999, ns<br>Foregut vs. Malpighian tubules: >0.9999, ns<br>Foregut vs. Ovary: >0.9999, ns<br>Foregut vs. Testis: 0.7482, ns<br>Midgut vs. Hindgut: 0.9723, ns<br>Midgut vs. Malpighian tubules: 0.9552, ns<br>Midgut vs. Ovary: 0.8284, ns<br>Midgut vs. Testis: >0.9999, ns<br>Hindgut vs. Malpighian tubules: >0.9999, ns<br>Hindgut vs. Ovary: >0.9999, ns<br>Hindgut vs. Testis: 0.8297, ns<br>Malpighian tubules vs. Ovary: >0.9999, ns<br>Malpighian tubules vs. Testis: 0.7823, ns<br>Ovary vs. Testis: 0.5668, ns

Experiment	Figure	Group	Statistics
	Figure. 5C	Stage-profile (*NPF*)	Test: One-way ANOVA + Tukey's test<br>F =34.43, df =7, 15, $P < 0.0001$ Sig.: ***<br>Adjusted P values for multiple comparisons are as follows:<br>Egg vs. Larvae I: 0.6527, ns<br>Egg vs. Larvae IIa: 0.9988, ns<br>Egg vs. Larvae IIb: 0.0033, **<br>Egg vs. Larvae III: 0.0067, **<br>Egg vs. Pupa a: 0.0015, **<br>Egg vs. Adult: 0.0651, ns<br>Larvae I vs. Larvae IIa: 0.8902, ns<br>Larvae I vs. Larvae IIb: 0.0649, ns<br>Larvae I vs. Larvae III: 0.1279, ns<br>Larvae I vs. Pupa a: <0.0001, ****<br>Larvae I vs. Adult: 0.0033, **<br>Larvae IIa vs. Larvae IIb: 0.0077, **<br>Larvae IIa vs. Larvae III: 0.0158, *<br>Larvae IIa vs. Pupa a: 0.0007, ***<br>Larvae IIa vs. Adult: 0.0279, *<br>Larvae IIb vs. Larvae III: 0.9996, ns<br>Larvae IIb vs. Pupa a: <0.0001, ****<br>Larvae IIb vs. Adult: <0.0001, ****<br>Larvae III vs. Pupa a: <0.0001, ****<br>Larvae III vs. Adult: <0.0001, ****<br>Pupa a vs. Adult: 0.405, ns
	Figure. 5D	Stage-profile (*NPFR*)	Test: One-way ANOVA + Tukey's test<br>F=46.04, df =7, 15, $P < 0.0001$ Sig.: ***<br>Adjusted P values for multiple comparisons are as follows:<br>Egg vs. Larvae I: 0.0039, **<br>Egg vs. Larvae IIa: <0.0001, ****<br>Egg vs. Larvae IIb: <0.0001, ****<br>Egg vs. Larvae III: <0.0001, ****<br>Egg vs. Pupa a: <0.0001, ****<br>Egg vs. Adult: <0.0001, ****<br>Larvae I vs. Larvae IIa: 0.001, ***<br>Larvae I vs. Larvae IIb: <0.0001, ****<br>Larvae I vs. Larvae III: <0.0001, ****<br>Larvae I vs. Pupa a: 0.0184, *<br>Larvae I vs. Adult: <0.0001, ****<br>Larvae IIa vs. Larvae IIb: 0.6014, ns<br>Larvae IIa vs. Larvae III: 0.0756, ns<br>Larvae IIa vs. Pupa a: 0.657, ns<br>Larvae IIa vs. Adult: 0.0991, ns<br>Larvae IIb vs. Larvae III: 0.7746, ns<br>Larvae IIb vs. Pupa a: 0.0521, ns<br>Larvae IIb vs. Adult: 0.8503, ns

Experiment	Figure	Group	Statistics
			Larvae III vs. Pupa a: 0.0039, **<br>Larvae III vs. Adult: >0.9999, ns<br>Pupa a vs. Adult: 0.0051, **
Expression under Heat stress	Figure. 6A	*NPF* (female)	Test: One-way ANOVA + Tukey's test<br>*F*=0.0718, df =2,6, *P* =0.9315<br>Sig.: ns
	Figure. 6B	*NPF* (male)	Test: One-way ANOVA + Tukey's test<br>*F*=3.654, df =2,9, *P* =0.0689<br>Sig.: ns
	Figure. 6C	*NPFR* (female)	Test: One-way ANOVA + Tukey's test<br>*F*=7.848, df =2,6, *P*=0.0211, Sig.: *<br>25 °C vs 36 °C: adjusted *P*=0.0349, Sig.: *<br>25 °C vs 39 °C: adjusted *P*=0.9908, Sig.: ns<br>36 °C vs 39 °C: adjusted *P*=0.0299, Sig.: *
	Figure. 6D	*NPFR* (male)	Test: One-way ANOVA + Tukey's test<br>*F*=16.86, df =2, 9, *P*=0.0009 Sig.: ***<br>25 °C vs 36 °C: adjusted *P*=0.0007, Sig.: ***<br>25 °C vs 39 °C: adjusted *P*=0.0147, Sig.: *<br>36 °C vs 39 °C: adjusted *P*=0.1310, Sig.: ns
Expression under starvation stress	Figure. 7	/	Test: Student's t-test<br>*NPF*_female: *t*=2.688, df =7, *P*=0.0312, Sig.: *<br>*NPF*_male: *t*=2.521, df =7, *P*=0.0397, Sig.: *<br>*NPFR*_female: *t*=2.584, df =7, *P*=0.0363, Sig.: *<br>*NPFR*_male: *t*=2.380, df =7, *P*=0.0489, Sig.: *
RNAi efficiency	Figure. 8	dsNPF vs. dsGFP (head)	Test: Student's t-test + Holm-Šídák correction<br>48h: *t*=6.814, df=5, adjusted *P*=0.001, Sig.: **<br>96h: *t*=9.939, df=6, adjusted *P*=0.0001, Sig.: ***<br>144h: *t*=8.779, df=8, adjusted *P*<0.0001, Sig.: ***<br>192h: *t*=6.871, df=10, adjusted *P*=0.0001, Sig.: ***
		dsNPFR vs. dsGFP (head)	Test: Student's t-test + Holm-Šídák correction<br>48h: *t*=2.037, df=5, adjusted *P*=0.1850, Sig.: ns<br>96h: *t*=5.992, df=4, adjusted *P*=0.0155, Sig.: *<br>144h: *t*=4.295, df=6, adjusted *P*=0.0155, Sig.: *<br>192h: *t*=0.3186, df=10, adjusted *P*=0.7566, Sig.: ns
		dsNPF vs. dsGFP (midgut)	Test: Student's t-test + Holm-Šídák correction<br>48h: *t*=17.7, df=4, adjusted *P*=0.0002, Sig.: ***<br>96h: *t*=11.52, df=4, adjusted *P*=0.0004, Sig.: ***<br>144h: *t*=14.94, df=4, adjusted *P*=0.0004, Sig.: ***<br>192h: *t*=13.17, df=4, adjusted *P*=0.0004, Sig.: ***
		dsNPFR vs. dsGFP (midgut)	Test: Student's t-test + Holm-Šídák correction<br>48h: *t*=6.468, df=7, adjusted *P*=0.0014, Sig.: **

Experiment	Figure	Group	Statistics
			96h: t=8.457, df=4, adjusted P=0.0032, Sig.: **<br>144h: t=4.361, df=4, adjusted P=0.0240, Sig.: *<br>192h: t=3.46, df=4, adjusted P=0.0258, Sig.: *
Food consumption	Figure. 9	Female	Test: One-way ANOVA + Dunnett's test<br>F=3.411, df =2, 86, P=0.0375, Sig.: *<br>dsGFP vs. dsNPF: adjusted P=0.0491, Sig.: *<br>dsGFP vs. dsNPFR: adjusted P=0.0463, Sig.: *
		Male	Test: One-way ANOVA + Dunnett's test<br>F=4.463, df =2, 87, P=0.0142, Sig.: *<br>dsGFP vs. dsNPF: adjusted P=0.0438, Sig.: *<br>dsGFP vs. dsNPFR: adjusted P=0.011, Sig.: *
Survival and reproduction	Figure. 10	Survival analysis	Test: log-rank test<br>Females: χ^2 = 3.935, df = 3, P = 0.2685, Sig.: ns<br>Males: χ^2 = 3.000, df = 3, P = 0.3916 Sig.: ns
		Egg production	Test: One-way ANOVA + Dunnett's test<br>F = 6.023, df = 3, 74, P = 0.0010 Sig.: *<br>dsGFP vs. Water: adjusted P=0.6834, Sig.: ns<br>dsGFP vs. dsNPF: adjusted P=0.0287, Sig.: *<br>dsGFP vs. dsNPFR: adjusted P=0.0595, Sig.: ns
Energy Metabolism	Figure. 11	/	Test: Student's t-test + Holm-Šídák correction<br>Glycogen (female): t=12.91, df=6, adjusted P=0.0000, Sig.: ***<br>Glycogen (male): t=13.38, df=6, adjusted P=0.0000, Sig.: ***<br>Trehalose (female): t=9.445, df=6, adjusted P=0.0002, Sig.: ***<br>Trehalose (male): t=6.195, df=6, adjusted P=0.0016, Sig.: **<br>Free fatty acid(female): t=0.2415, df=6, adjusted P=0.8172, Sig.: ns<br>Free fatty acid(male): t=0.1435, df=6, adjusted P=0.8906, Sig.: ns
